# This is our rhythm: academic becoming and realignment in deaf space

**DOI:** 10.1093/jdsade/enaf061

**Published:** 2025-12-08

**Authors:** Maartje De Meulder, Joseph J Murray, Annelies Kusters

**Affiliations:** HU University of Applied Sciences, Utrecht, The Netherlands; Gallaudet University, Washington, D.C., United States; Heriot-Watt University, Edinburgh, United Kingdom

## Abstract

Deaf scholars have long worked at the margins of academic institutions not designed for them. Designated deaf academic spaces—where deaf ways of knowing, teaching, and communicating are centered—remain rare. This study explores what becomes possible when such a space exists, presenting *Dr Deaf* as a case study. Drawing on interviews with participants and teachers, we show how deaf epistemologies and pedagogies are enacted through *cross-stage responsibility* and *academic becoming* through *re-alignment* of deaf participants and teachers. We also identify a distinct *deaf rhythm* that emerges in this space. At the same time, we recognize that these practices are not experienced or valued equally by all participants and teachers: needs, priorities, and ways of engaging differ, and *Dr Deaf’s* approaches may not resonate for all. Yet its values offer a flexible framework for imagining and sustaining other deaf academic and broader educational spaces.

Designated deaf academic spaces—where deaf academics’ ways of knowing, teaching, and learning are the norm—remain rare. In most academic spaces, deaf academics are in the minority—often working in hearing departments, teaching hearing students (often future sign language interpreters), and relying on interpreter-mediated communication. Deaf students, in turn, are rarely taught by deaf academics and typically also experience a large part of academic life through the lens of sign language interpretation or other forms of accommodation.

This paper explores what an academic space can look like when built on deaf scholars’ terms. Using *Dr Deaf* as a case, we examine how academic structures, pedagogies, and interactions are realigned to center deaf epistemologies, language practices, and values. *Dr Deaf* is a transnational initiative where International Sign (IS) is used as the shared language of communication. Drawing on interviews with participants and teachers, we examine how this deaf academic space is enacted in practice—and what becomes possible when the usual structures are realigned.

We introduce the concept of *cross-stage responsibility* to describe the horizontal and vertical mentoring practices that characterize *Dr Deaf*, and frame it as a site of academic becoming—where both teachers and participants shape and affirm their identities as *deaf* academics. Central to all this is the notion of realignment: deaf teachers teaching deaf students through direct communication in IS, and the reconfiguration of authority, access, interaction, and knowledge production in ways that center deaf perspectives. Alongside this realignment runs a distinct *deaf rhythm—*a shared tempo of interaction, reflection, and relationality that emerges in this deaf academic space. At the same time, we show that what works well for some participants can pose challenges for others, and that differences in privilege and diversity within the group shape participation. Through this case, we consider the broader value and fragility of deaf academic spaces, and reflect how insights from *Dr Deaf* might inform other deaf academic and educational spaces.

## Background and theoretical framework

Deaf scholars have long worked at the margins of institutions not designed for them (Haualand, 2017; Kusters et al., 2017)—a reality that has been documented in research on inclusive higher education policies (Hendry et al., 2020; Kermit & Holiman, 2018; Robinson & Henner, 2018; Woodcock et al., 2007) and in accounts from deaf academics and students navigating higher education institutions (Chua et al., 2022; Garberoglio et al., 2019; Majocha et al., 2018; Moges-Riedel, 2020; O’Brien, 2019, 2020; Smith & Andrews, 2015; Stapleton, 2014, 2015; Yabe, 2024). These studies point to the way structural inaccessibility, audism, token inclusion, and institutional norms continue to shape and often undermine deaf academic lived experiences and career advancement. Some of the studies highlighted how these barriers are compounded or reshaped by racial dynamics, producing uneven experiences and opportunities within academic spaces more broadly.

Designated deaf academic spaces—especially those that are transnational—remain rare. While spaces such as the biennial Deaf Academics Conferences or institutions such as Gallaudet University and National Technical Institute for the Deaf (NTID) in the USA and Tsukuba University of Technology in Japan exist, for many deaf academics sustained access to deaf academic space is limited.

There are also a few non-academic transnational deaf *learning* spaces that cater to deaf participants. An example is Frontrunners, a nine-month international deaf leadership course for deaf youth held at Castberggård Folk High School in Denmark. At Frontrunners, IS is the language of instruction and communication. Ål Folk High School in Norway, where the *Dr Deaf* sessions are being held, also provides courses for international deaf participants, for example on sign language theatre.

It is important to distinguish between transnational academic *deaf* spaces and transnational academic *signing* spaces, which are sometimes conflated or treated as equivalent. Academic *signing* spaces are typically mixed deaf-hearing spaces where IS (and sometimes English) is used as the language of instruction and conversation. One example is the European Master in Sign Language Interpreting (EUMASLI), which admits both deaf and hearing interpreters through a selective application process and uses IS as the language of instruction. A more recent example is the Summer School of Sign Language Studies for deaf and hearing advanced students and early career researchers, hosted by Humboldt Universität Berlin, with IS as the sole language of instruction. While such spaces are valuable for promoting academic discourse in IS and enabling deaf and hearing participants to interact directly, they are different from *deaf* academic spaces, which are shaped by the values and modes of interaction explored in this article.

This paper examines such a deaf academic space, using *Dr Deaf* as a case study. At the heart of our analysis is the concept of deaf epistemologies—deaf ways of knowing, learning, and meaning-making—that challenge the hearing-centric norms of mainstream academia (Hauser et al., 2010; Ladd, 2003). *Dr Deaf* makes space for these epistemologies to emerge not only in the use of IS or the presence of deaf teachers, but also in the rhythms of interaction, the culture of mutual support and collective wisdom, and ongoing calibration. In this sense, *Dr Deaf* offers one possible form of a *deaf academic space* in its own right (Gulliver, 2017; O’Brien & Emery, 2014).

Building on deaf pedagogies (Kusters, 2017; Ladd, 2022), we understand pedagogies within *Dr Deaf* as relational, dialogical, and collective. Teachers are often former *Dr Deaf* participants (and some continue to participate when not in a teaching role), participate in peer feedback groups, join dinners, and social events. Many of the deaf pedagogical values that Ladd (2022) described are found in the *Dr Deaf space*: moral guidance, information-sharing, and high expectations, while aiming to create safe deaf spaces. Expanding on Marieke Kusters’ (2017) concept of *intergenerational responsibility* in deaf pedagogies, we introduce the notion of *cross-stage responsibility* to describe how at *Dr Deaf*, deaf academics at different career stages actively support one another. These academic career stages do not necessarily map onto age or generations as in Marieke Kusters’ work: at *Dr Deaf*, PhD participants may be older than professors, and teachers may be younger than some participants. Rather than reinforcing age-based models of expertise, cross-stage responsibility reflects a shared commitment to mutual growth. This orientation echoes a broader ethos of “giving back” within Deaf communities (De Meulder, 2007; Ladd, 2003).

This does not mean that participating in such spaces requires no effort. For example, for new signers of IS (De Meulder, 2018; Kusters, forthcoming), communication in international deaf spaces may be challenging. While it may deter some from participating, it is a challenge participants who have taken the step seem to accept, contrasting it with the “illusion of inclusion” in mainstream educational environments (Russell, 2021). In these environments, interpreting services may be provided, but deaf learners remain on the periphery and must carry the additional cognitive and emotional labor of navigating access through interpreters, a phenomenon we refer to as “interpreter tax” (De Meulder & Haualand, 2021; O’Brien et al., 2023; Pirone et al., 2018) and “gaze work” (De Meulder & Stone, 2024). For deaf scholars of color, this labor is often complicated and intensified by race and gender dynamics (Hou and Moges, 2023).

Deaf lecturers use a range of strategies to foster understanding in multilingual, multimodal classrooms. They combine sign languages, fingerspelling, mouthing, and written languages, often anchoring meaning via PowerPoint slides, whiteboards, or blackboards (Holmström & Schönström, 2018). Technology, too, plays a role: for example, AI on mobile phones assists with translation. Communication is frequently co-constructed—teachers expanding on each other’s input, or participants supporting one another during activities (Kusters, 2024). These strategies are crucial in classrooms with deaf participants from diverse linguistic backgrounds (Duggan et al., 2023).

Finally, *Dr Deaf* must be understood within the broader ecology of transnational deaf academic and professional mobility (Hou & Ali, 2024; Kusters, 2024a). Deaf professional mobility refers to the movement of deaf individuals across local, national, and transnational spaces in pursuit of education, employment, advocacy, and scholarly engagement. It is shaped by uneven access to sign language rights, educational opportunities, and professional recognition, and is often driven by the need to seek more enabling environments elsewhere. Such mobility, such as to the *Dr Deaf* sessions in Norway—is not only geographic; it also involves navigating linguistic and ideological terrains—for example, adjusting to the use of IS, adapting to majority-white spaces, to unfamiliar norms, or confronting assumptions about deaf professionalism. These experiences are shaped by intersecting factors such as race, gender, class, and migration status, which influence whose mobility is facilitated, and whose is constrained, and whose contributions are most enabled and recognized in international professional spaces.

Deaf professionals from different backgrounds often act as cultural and linguistic brokers, drawing on diverse repertoires and experiences to establish credibility, build networks, and create pathways for others. At the same time, their mobility is constrained by systemic inequalities, including visa regimes, access to funding for travel, lack of networks/connections that can point the way to deaf professional spaces, and normative expectations shaped by hearing-centric institutions. Understanding deaf professional mobility thus requires attention not only to individual trajectories but also to the broader structures and ideologies that enable or restrict the circulation of deaf knowledge and expertise across borders.

## Origins and development of *Dr Deaf*

*Dr Deaf* was founded in 2018 as a network, an idea, and a space. On the one hand, it was created in response to the need for deaf academic spaces outside of and complementary to the biennial Deaf Academics Conferences, which usually attract over 100 participants. There was a need for spaces that were smaller in scale, more frequent in timing, and more focused in purpose. On the other hand, *Dr Deaf* also emerged in response to the persistent inaccessibility—or only partial accessibility—of many university-based academic resources and training opportunities for deaf students. The idea was closely tied to the Ål folkehøyskole and workshop center for deaf people, located in a mountain village in Norway, 3 hr from Oslo. Joe lives in Ål and his knowledge of the school, combined with its deaf-owned infrastructure and physical facilities, provided an ideal setting for such a space—both practically and ideologically. The Ål folkehøyskole, which hosts the *Dr Deaf* sessions and receives funding from the Norwegian government, allows to organize the *Dr Deaf* sessions at relatively affordable rates. Ål also offers a signing environment and a model of deaf institutional ownership that resonated with *Dr Deaf’s* aims.

Joe initiated the first *Dr Deaf* session in 2018. Annelies and Maartje were initially invited as guest teachers, and were asked in 2021 by Joe to consist a core team (Annelies left the core team in 2025). We knew each other as friends and academic colleagues who met each other on the deaf conferences circuit, and had worked and published together. Our collaboration for *Dr Deaf* emerged through many informal conversations and a shared commitment to developing and supporting future generations of deaf academics and strengthening pathways to career success for current academics.

In the early stages of our academic journeys, we had no space like *Dr Deaf*—much of what we learned about academic life, we figured out on our own, or from our peers. That experience was a key motivation for our work for *Dr Deaf*: to make the path a little clearer and smoother for the current and next generation of deaf scholars. Currently, we all three hold permanent roles in institutions where we teach Deaf Studies and/or sign language interpreter courses. This stable institutional footing gives us a structural privilege and institutional knowledge to contribute to this space.

Since 2018, *Dr Deaf* has organized 14 five-day sessions and welcomed over 300 participants from six continents (counting repeat participation). At times, sessions run in parallel, such as *PhDeaf* and the Writing Retreat (both discussed below). The average number of participants in the sessions is 20. We do not use an application or selection process to determine who can participate. Admission is open to all interested individuals on a first-come, first-served basis upon registration and payment of the participation fee. There are no screenings or eligibility requirements (apart from being deaf and in academe). We acknowledge, though, that people often participate because they learn about *Dr Deaf* in our or their networks and the networks of guest teachers and previous participants, which means that white deaf Europeans and North Americans have been overrepresented. The majority of our participants have been women, mirroring gendered imbalances among younger academics in these populations.

From the beginning, *Dr Deaf* has been grounded in several core values:

- The creation of signing, deaf-centered mentoring spaces;
- The development of deaf academic peer networks;
- Encouraging more deaf PhDs;
- Fostering academic IS spaces, while welcoming new signers of IS; and
- Providing training for deaf scholars at various stages in their careers.

The all-deaf, signing space is a crucial aspect of *Dr Deaf*. There are no hearing participants. The only interpreters present are deaf interpreters working with deafblind participants, using ProTactile and tactile signing (van der Mark, 2023) or sitting positioned in participants’ visual fields. The absence of any other interpreters allows for largely unmediated conversations and learning environments (apart from contexts where brokering happens)—free from the constant management of hearing-deaf differences and spoken-sign interpreting that are a regular part of deaf academic lives in hearing academic spaces, and significantly contribute to impostor syndrome (Chua et al., 2022). These experiences in hearing academic spaces include managing hearing fragility, audism, know-your-place aggression, and deaf tax (Aldalur et al., 2022; O’Connell, 2021).

The use of IS has been central to the success of *Dr Deaf*. IS use within *Dr Deaf* reflects a shared commitment to the production of academic spaces where no single national sign language dominates. IS is thus a socially and morally situated choice (Green, 2014; Moriarty & Kusters, 2021). While conventional versions of IS are used in the *Dr Deaf* space, we also use IS in the sense of calibration: teachers and participants adjust their signing to specific interlocutors. This practice of switching between conventional IS and calibration is typical for educational spaces where IS is used (Kusters, 2024).

A key part of the program is the *PhDeaf* sessions, with which *Dr Deaf* started in 2018. These sessions are designed to facilitate the academic journeys of aspiring and current deaf PhD participants across all disciplines. Topics include academic writing, identifying research goals, conducting literature reviews, applying for a PhD, working with supervisors, managing accessibility and interpreter support, and more. Since 2018, over 40 *Dr Deaf* participants have entered PhD programs, and several are now close to completion.

Another key part of the *Dr Deaf* brand is the Writing Retreats, held twice a year. These are open to deaf academics at different career levels—from master’s participants to full professors—and include extended writing time, peer review groups, and daily workshops on academic publishing, writing, time management, use of AI tools, and more. In December 2025, we organize the 8th Writing Retreat. We have also organized one thematic workshop in 2022, on qualitative research methods. In October 2025, the first Professor Deaf session will be held, aimed at academics post-PhD, at mid-senior career level, working in a university or research setting.

The *Dr Deaf* teachers and facilitators are all deaf academics with PhDs who are happy to teach in IS. The core team usually teaches themselves and, to complement the team, invite guest teachers, aiming to ensure diversity in career stage, disciplinary background, language repertoires, geographical context, and ethnicity across different *Dr Deaf* sessions. In practice, however, selections have also been shaped by pragmatic considerations: we have prioritized demonstrated IS and teaching experience; invitations have tended to draw on our existing networks; and funder constraints (e.g., possibility to cover several long-haul invitations). We regularly invite former participants, including early career researchers with recently obtained PhDs, to grow the pool of *Dr Deaf* teachers and to strengthen *Dr Deaf’s* continuity. Each workshop or session typically includes at least three teachers, when sessions run in parallel (e.g., PhDeaf and Writing Retreat), there can be up to six.

## Methodology

This study draws on nine participant interviews and 11 teacher interviews, analyzed through a phenomenological lens to understand how deaf academics experience and make sense of being in a deaf academic space. Our focus is not so much on individual narratives as on similarities in how participants describe the significance of this deaf academic space in relation to their academic journeys.

### Participant interviews

The nine participant interviews were conducted by the first author in the first half of 2025. These semi-structured interviews were held online and conducted in IS, with durations ranging from 30 min to 1 hr. Participants were selected through purposeful sampling to ensure a diverse sample in terms of gender, ethnicity, career stage, disciplinary background, and geographical location, including both first-time and returning participants. Table 1 provides an overview of participant characteristics. Questions explored participants’ motivations for attending *Dr Deaf*, their expectations and challenges at the time, and their experiences within the *Dr Deaf* space, including any challenges. Questions also addressed how participants navigated the deaf-only, direct teaching, and IS environment, and how *Dr Deaf* compared to other academic contexts. Participants were also asked about the impact of *Dr Deaf* on their academic journeys, what advice they would offer to future attendees, and how they saw the future of *Dr Deaf*.

**Table 1:** Overview of participant characteristics.

	**Name**	**Country**	**Field**	**Career stage at the time of the interview**	**Self-described racial/ethnic background** ^1^
1	Phoebe Tay	Singapore	Applied linguistics	PhD student (near completion)	Asian
2	Liona Paulus	Germany	Linguistics and sign language interpreting studies	Professor	White
3	Franklin Jones Jr.	USA	Education	PhD student	Black
4	Naiara Larrakoetxea	Spain (Basque Country)	Political sciences	PhD student (near completion)	White
5	Kali Lacy	USA	Data science	Finishing MA, considering a PhD	Asian
6	Alessio Di Renzo	Italy	Documentation studies, linguistics and literature	PhD student (defending soon)	White
7	Alexandra Dongal	France	Sign language translation	Finishing MA, considering a PhD	Carribean
8	Robin Angelini	Austria	Human-computer interaction	PhD student	White
9	Maija Koivisto	Finland	Deaf Studies/interdisciplinary	PhD student (defending soon)	White

### Teacher interviews

The 11 teacher interviews were conducted by the three authors (divided among us) during the first half of 2025. We did not apply sampling for the teacher group: all those who had taught at *Dr Deaf* by the time of data collection were interviewed. We developed and agreed on a shared set of questions to ensure consistency across interviews. Most interviews were held online, while two took place in person. Interviews were conducted in IS, ASL, and BSL, depending on interviewees’ language preferences and relationship to the researcher. Most teachers were interviewed individually, while two interviews were conducted in pairs. Several of the interviewees had previously attended *Dr Deaf* as participants and/or continue to attend as participants when not in a teaching role. Table 2 gives an overview of teacher characteristics. Teacher interviews focused on their motivations for teaching at *Dr Deaf*, their perceptions of what makes *Dr Deaf* different from other academic training programs, and the value of *Dr Deaf* as a deaf space, interpreter-free space, and IS space. Questions further addressed teaching in a multilingual environment, experiences with IS, and teaching strategies. Teachers also reflected on how *Dr Deaf* has shaped their thinking about deaf education, inclusion, and their own professional development, and shared thoughts about the future of *Dr Deaf*.

**Table 2:** Overview of teacher characteristics.

	**Name**	**Country of professional affiliation**	**Field**	**Career stage**	**Ethnicity**
1	Hilde Haualand	Norway	SSH	Professor	White
2	Ingela Holmström	Sweden	SSH	Professor	White
3	Peter Hauser	USA	STEM	Professor	White
4	Octavian Robinson	USA	SSH	Associate Professor	White
5	John Pirone	USA	SSH	Assistant Professor	White
6	Alicia Wooten	USA	STEM	Associate Professor	Biracial Japanese- American
7	Katherine Rogers	UK	SSH	Associate Professor	White
8	Nora Duggan	Sweden	SSH	Assistant Professor	White
9	Okan Kubus	Germany	SSH	Professor	PoC
10	Robert Adam	UK	SSH	Associate Professor	White
11	Mette Sommer	UK	SSH	Assistant Professor	White

SSH: Social Sciences and Humanities; STEM: Science, Technology, Engineering, Mathematics.

### Author positionalities and focus

The interviews were conducted by the three authors, who also form the core team behind *Dr Deaf* and are involved as teachers. We are white, senior academics based in Europe and affiliated to universities in the Netherlands, the United Kingdom, and the United States, with disciplinary backgrounds in the social sciences and humanities. Maartje and Annelies are in their early forties, while Joe is in his early fifties. We obtained our PhD degrees between 2007 and 2016. We identify as female (Maartje and Annelies) and male (Joe ). We are fluent in IS and English, using both languages among our working languages and languages used at home.

Us being the core team behind *Dr Deaf* for several years shaped our access to participants and teachers, and the interpretative lens through which we analyzed the data. This positionality brings both strengths and limitations. On the one hand, it enabled contextual understanding; on the other, it shaped the design of this study, the framing of questions, what participants told us (and especially what they did not), and how we interpreted their narratives. In addition, as initiators and organizers of *Dr Deaf*, we hold positions of power and influence that may shape who feels welcome or comfortable participating in *Dr Deaf*. We acknowledge the probability that interview participants have self-censored due to concerns that negative feedback could affect how they feel about future involvement as teachers or participants in *Dr Deaf*. We explicitly also asked about challenges inherent in and critical reflections about *Dr Deaf*, but we acknowledge that interviewees probably felt they could not be too critical. We acknowledge that our positionalities will have introduced unconscious biases or assumptions—particularly in relation to race and ethnicity, geography, socio-economic status, and disciplinary backgrounds. Likewise, in our selection of interviewees for this study, it is possible that we unconsciously prioritized participants with whom we have more established or positive relationships. The sum of these dynamics may have contributed to the interview data’s largely positive tone and its emphasis on what we share as deaf academics, rather than on points of difference—something we consider a limitation of the study. We therefore complemented some of the interview data below with anecdotal evidence from informal conversations with participants and teachers, as well as from *Dr Deaf* evaluation sessions, which also highlighted challenges that participants did not raise in the interviews.

### Data analysis

Interview data were analyzed thematically by the first author through an iterative and interpretive process. This involved repeated viewing of interview recordings, close attention to notes made by co-interviewers, and ongoing discussion among the authors. Excerpts cited in this article were translated into English by the first author and checked by participants.

Due to the nature of our data, our analysis centers deaf academic experience as a *shared* point of departure, without treating it as uniform. Our primary aim was to explore the potential of deaf-led spaces like *Dr Deaf*. At the same time, we remain attentive to how participants’ experiences were shaped by other dimensions of identity and context, and how these intersected with being deaf. We allowed these themes to emerge inductively from participants’ narratives, as well as from the above-mentioned anecdotal information.

## Findings

### *Dr Deaf* as a deaf space with a deaf rhythm

Participants and teachers were unanimous about the importance of *Dr Deaf* being a *deaf* space—not just a signing space. This deaf space is shaped by deaf ways of being, thinking, and interacting. For several participants, *Dr Deaf* was their first experience of being in an academic environment where deaf norms and epistemologies were the standard rather than the exception. Interviewees often described their experiences of *Dr Deaf* through metaphor—a finding that aligns with previous research on how metaphor functions in deaf narratives to express complex, layered experiences (Kusters & De Meulder, 2013, Kusters & De Meulder, 2019; Kusters, forthcoming). For example, Alessio described the entrance as luxurious in comparison to hearing academic spaces, using the metaphor of a red carpet:

*The most important thing for me was how directly accessible Dr Deaf was. With other academic events you usually must jump through several hoops just to get in—and even then, it’s uncertain whether it will be worth the effort at all. With Dr Deaf, the doors just opened. You’re welcomed in straight away—it felt like stepping onto a red carpet.*

In this deaf space, direct and unmediated interaction is possible for most of the participants, which is seen as conducive to genuine academic exchanges. Participants said they are often tired of the need to “teach” and “explain” deaf experiences in hearing academic spaces. Franklin: “I just want to sit down with someone and have an academic conversation without having to teach them first. This is something that happens at *Dr Deaf.*” Hilde, one of the teachers who also regularly joins as a participant, echoed this sense of mutual understanding: “Many of our baggage is similar. There is no need to explain. You can put the baggage aside and go straight to the core.” Importantly, participants felt that this deaf space prioritized *their* learning experiences *as a deaf person*. This may sound straightforward but is not the experience many deaf participants have in their university environments, where inclusion often feels one-sided. Robin: “It’s always the same in these [mixed, hearing-dominant] spaces—hearing participants learn, while deaf participants just sit there and don’t.”

These exchanges and learning experiences unfold at a pace participants described as a “deaf rhythm”—a way of working, thinking, and being that aligns with deaf experiences rather than defaulting to hearing academic norms. It is experienced as a natural tempo grounded in signed communication and deaf ways of being. Robin described it as follows:

*The pace in a deaf space is different—we’re all signing, I can ask a question when I want to and I don’t need to wait for the interpreter to catch up or be annoyed that after my question everyone is silent [...] when I ask something there always is another contribution immediately, and we all add to each other.*

Deaf ways of attention-getting and turn-taking are central to deaf rhythm. Teachers also adapted their methods to align with this rhythm: allowing participants time to translate slides with their phones’ AI or adjusting signing to accommodate varying fluency in IS or English. This idea of “deaf rhythm” resonates with the broader concept of “crip time” (Samuels, 2017)—the use and experience of time as a disabled person.

This rhythm is supposed to be flexible and responsive to individual needs, with variations in pace and access. Yet, while celebrated by many sighted deaf participants, it does not always work in the same way for deafblind participants. In a group where sighted deaf participants are the majority, their preferred pace and interactional style can easily set the norm—for instance, celebrating the absence of interpreters. Deafblind participants may move within a different rhythm if they work with interpreters. This calls for a conscious tuning of the group’s tempo so that the rhythm is inclusive of the ways deafblind participants navigate space and interaction. When interpreters are not present, accessibility for deafblind participants becomes a shared responsibility within the group. For example, teachers wear plain clothing that contrasts with their skin color, and participants, when making a comment or asking a question, get up and position themselves at the front instead of signing from their seats. This demands a collective attentiveness that pushes against entrenched norms, also often present in deaf spaces, of vidism and distantism (Clark, 2021).

Since an important part of the *Dr Deaf* sessions are the writing retreats, the significance of writing in this deaf space was also highlighted. Writing was described as an intimate and vulnerable act, especially for those who had been either underestimated or excessively praised. Octavian, one of the teachers, recounted:

*Growing up we are constantly compared to hearing kids. I’ve had teachers and interpreters tell me, “Wow, your English is really at hearing level,” as if that is the highest praise. With other deaf people, I feel more equal. We’re coming from a shared experience, and that makes it easier to write together, give feedback, and be open. The absence of judgment, and of that constant comparison to hearing norms, makes a huge difference.*

Okan, one of the teachers, described a similar experience of how the *Dr Deaf* space supported their writing: “seeing someone else do it makes me feel there is an opening, and I become open to exploring themes I used to feel nervous about.” At the same time, differences in language knowledge and writing experience could heighten impostor syndrome or limit the possibility to engage with the English-language slides used during the *Dr Deaf* workshops. Okan pointed out that for some, transitioning to English is hard work. *Dr Deaf* participants come from countries where they write in different languages, often not English. Okan felt that English knowledge is also associated with a certain privilege and status. They felt that teachers who are more Western-minded, more privileged, fluent in English, and have more publications [in English] tend to receive more attention and respect. Meanwhile, those with weaker English skills and fewer publications may feel overlooked or undervalued.

Most participants were very clear about the need to keep this space a deaf space and couldn’t imagine sharing it with hearing—even signing—participants. Alexandra recounted:

*We already spend most of our time in hearing-majority educational spaces. If Dr Deaf started to allow hearing, even signing, participants, I think many deaf participants wouldn’t want to come anymore. This is a deaf space, and it needs to stay that way. Deaf space is fragile. It is my space. Don’t touch it.*

John, one of the teachers, described *Dr Deaf* as his home, saying “admitting hearing people would be like having strangers entering my home.” Octavian shared a similar sentiment: “90 percent of my working life is in the hearing world: hearing colleagues, participants, interpreters—it just goes on and on. Seldom do I have an opportunity to be in deaf academic spaces.”

*Dr Deaf* was also praised as an interdisciplinary academic deaf space. Octavian: “*Dr Deaf* participants are serious about their work and their projects. There is a very good intellectual energy. We are here to do something.” Teachers also appreciated how *Dr Deaf* created a shared pool of deaf academic knowledge to draw on in their teaching. Mette described how this enabled real-time collaboration, even during parallel sessions for separate groups, such as the writing retreat session and the PhDeaf session: “I like the flexibility of *Dr Deaf*. When we might be teaching about getting funding, we could just invite someone from the writing group to come and share their experience. It’s a collective.”

### Becoming a *deaf* academic: deaf epistemologies in action

In both participant and teacher interviews, *Dr Deaf* was described as a space of deaf academic *becoming* through shared exploration of research methodology and positionality. Naiara, a participant who attended every *Dr Deaf* session in its first five years, noted: “*Dr Deaf* is my alma mater—it’s where I learned how to be a deaf academic. I cannot separate my positionality as a deaf person from my researcher positionality. *Dr Deaf* taught me how to combine the two.”

The *Dr Deaf* space allows for explorations of deaf epistemologies in ways that participants felt could not happen in hearing academic spaces. John described this as “having access to *Deaf epistemologies in action—*our ways of knowing and meaning-making within the academic world.” Both participants and teachers noted it is easy to forget how to be a deaf academic when working full-time in a hearing academic world. Peter: “some deaf academics become hearing academics, thinking that’s the ‘right’ way to be. *Dr Deaf* helps them to see another way.”

The presence of deaf teachers added an additional layer of significance. While most participants had experience being taught by a deaf teacher at some point in their schooling, for some, being taught by a deaf *academic* and seeing academic concepts explained in sign, was a first-time experience. Maija linked this to deaf epistemologies: “A hearing person, even if they know how to sign, would never explain it in the same way. There is always something missing.” Mette emphasized the importance of representation: “At *Dr Deaf*, having a deaf teacher is so important—they are sharing their journey.”

In addition to learning from deaf academics, participants also emphasized the value of peer feedback within a shared linguistic and cultural framework. Alessio, who was at the time of the interview working on a PhD in sign linguistics, recalled how meaningful it was to receive peer feedback from other deaf signers on his writing:

*I was in a feedback group with two people who aren’t linguists, but because they know sign language, they understood my work, and we could communicate directly. I remember one group member kept asking me questions, so in a way, it became as much a learning experience for them as it was for me—even though their role was to give me feedback. I was more than happy to be part of that exchange.*

In contrast, participants feel that in hearing spaces, their questions were often misunderstood, dismissed, or seen as “weird.” Robin: “when I ask a methodological question about how to apply something to interviews with deaf signers for example, they say good question, I’ve never thought about this before. It often leaves me unsatisfied.”

What emerges is a clear contrast between hearing academic spaces where deaf academics often feel the need to adapt or translate themselves, and international spaces like *Dr Deaf*, where their ways of thinking, knowing, and being—deaf epistemologies and ontologies—are seen as the norm, and where they discovered deaf ways of being an academic, even if their individual journeys and experiences were also shaped by other aspects of their positionalities (which we discuss below).

### *Dr Deaf* as community and belonging: “I am not alone”

A central and consistent theme across all interviews was the discovery of an international community of deaf academics when attending *Dr Deaf*. Both participants and teachers spoke with emotion about encountering (for some participants for the first time), a space with so many deaf academics, at different career stages, working in different disciplines, and in different countries. This gave them access to imagined deaf academic futures, especially for people coming from countries where there were only a few deaf academics. Naiara recounted:

*What I remember most from that first workshop is that I thought wow, there are so many deaf PhD researchers or deaf people thinking about doing a PhD—I always thought it was just me. All these different fields and different career stages—but all in the same space.*

Mette: “As a student at *Dr Deaf*, I used to think, ‘I could never be like them’. It’s not DEAF-SAME—and that’s interesting too: the *Dr Deaf* space shows the different levels of deaf people in academia.” For her, and for others, this meant both feeling a disconnect from more experienced peers and gaining a clear goal to work toward. Many described this experience of finding an academic deaf community as transformative, particularly in relation to academic becoming. Seeing so many deaf academics in one place made participants realize they could *belong* in academic spaces. Robin recalled, “*Dr Deaf* showed me I could become a researcher—that there was a place and a network for me.” Nora, a teacher and former participant, described it as “a big realization: I can do this. I can belong in academic spaces.” The presence of role models—especially those with non-linear paths into academia—was repeatedly mentioned as a source of inspiration: people who had started PhDs later in life, combined academic work with activism, or navigated research alongside other careers—a common experience for minority academics who are often juggling multiple roles. Several participants cited *Dr Deaf* as the reason they started their PhDs after being initially uncertain. As Naiara put it: “Seeing so many deaf people successfully doing research convinced me I could do it too.”

But the influence of *Dr Deaf* went beyond just *starting* a PhD. It helped participants to *keep going*, to navigate challenges, and to *finish*. Participants and teachers used different metaphors to describe this sustaining power. Many spoke of *Dr Deaf* as a battery charger or a shot of energy—a regular and vital boost needed to survive in hearing-dominated academic spaces. Others described it as the satisfaction of receiving something long craved, finally within reach.

A different set of metaphors spoke to the collective aspect of the experience. Maija saw deaf academics as sailing boats navigating alone, but always aware of each other’s presence—a vision that echoes Bahan’s (1994) metaphor of the deaf community as a harbor. Phoebe captured this spirit through a visual description of participants taking each other’s hands and pushing one another forward, over the limits. Octavian described it as climbing a ladder while pulling others up: “When I climb, I don’t want to climb alone. That is the energy of *Dr Deaf*.”

The importance of networking came up repeatedly. This network has been consolidated and expanded in the seven years since the first *Dr Deaf* session. Liona remarked: “it’s beautiful to see how at international conferences people greet each other with ‘oh yes I met you in Ål!’” Mette reminisces about her experience at the first PhDeaf workshop:

*The networking was amazing. I saw faces I’d never seen before—people from diverse backgrounds, even some from STEM. And later, at the second workshop, I saw how some participants who were almost done with their PhDs kept coming back to support others who were earlier in their PhD career. There was no arrogance.*

This sense of community is not an accidental by-product of *Dr Deaf*. The *Dr Deaf* sessions intentionally include community-building activities, both within and outside formal teaching times. These activities include peer feedback groups in which teachers participate alongside participants, submitting their own work for feedback. John, a teacher and participant, marvelled at this. “I’m being asked to comment on a famous academic’s draft?”. The sessions are designed with ample social time, and both intentionally and organically developed collective rituals borrowed from deaf cultural traditions. This includes outdoor group activities in which participants must pitch in via small groups—for example, getting the food, setting up the fire, cleaning up afterwards—morning reflections from participants or teachers which promote a sense of openness, and evening storytelling or game activities that emerge in social spaces.

**Figure 1:**
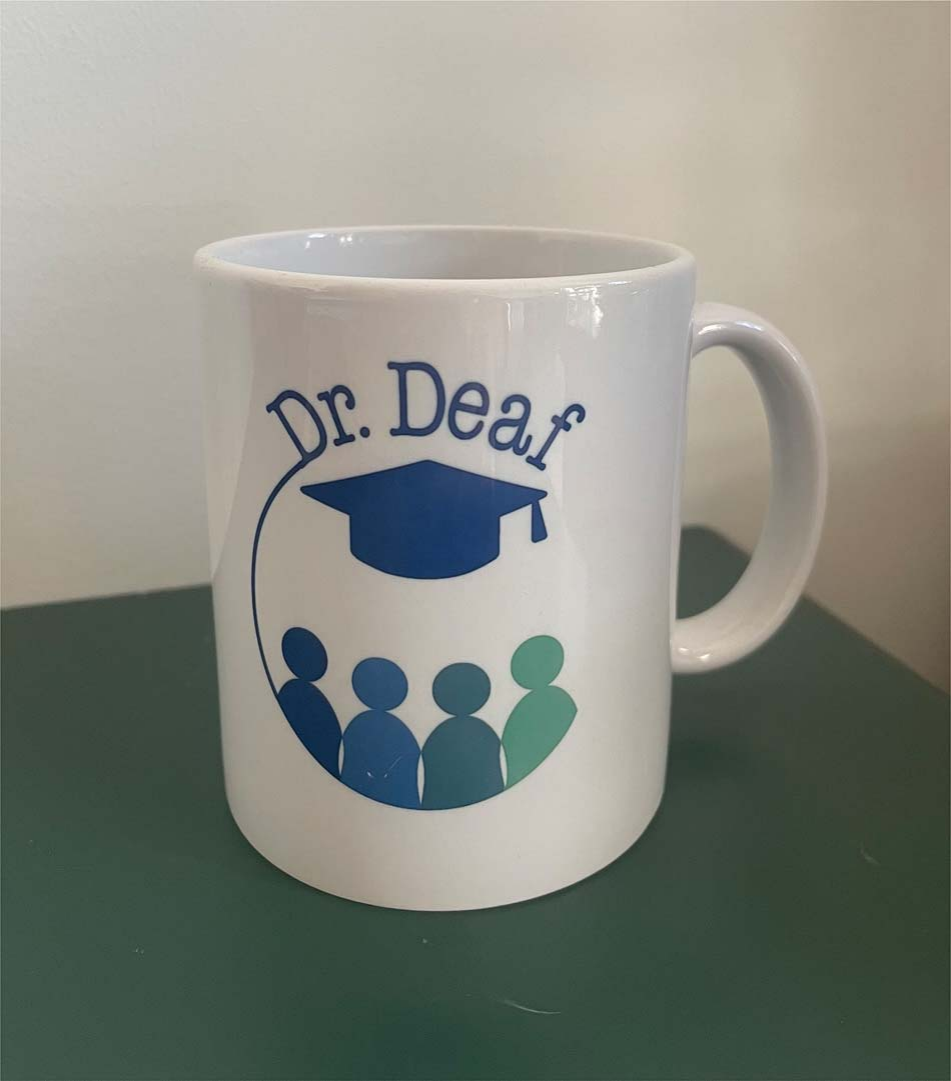
*Dr Deaf* mug. A ceramic mug featuring the *Dr Deaf* logo with a graduation cap above a row of stylized human figures. Logo designed by Marta Morgado.

Finally, in this space, recognition and encouragement also mattered. In 2023, the *Dr Deaf* core team started a tradition of handing out a designated *Dr Deaf* mug with the *Dr Deaf* logo on it (see Figure 1), to those participants who successfully received a PhD. The team presented this as a mug “money cannot buy.” Nora described how receiving the mug after her PhD defense meant a great deal to her—a small but powerful symbol of belonging and affirmation in a space she now contributes to as a teacher.

Yet a recurrent concern in relation to belonging and networks was the limited geographical and racial diversity within the participant *and* teacher group. While *Dr Deaf* aims to bring together participants and teachers from different disciplines, backgrounds, and countries, some noted that access remains uneven. As Maija put it: “Our research interests are diverse but our backgrounds not so much—we are already privileged because we can attend.” Financial barriers were also mentioned. The expense of traveling to Norway was a significant burden for some. Not all participants received university support or external funding, and several paid out of pocket. These financial constraints reflect broader global inequalities in access to academic networks. Participants from the Majority World, in particular, face limited opportunities to join international programs like *Dr Deaf* due to lack of institutional support, visa barriers, and restricted funding, but also due to limited access to transnational networks, which are often crucial for learning about such opportunities through word-of-mouth recommendation and encouragement (Hou & Ali, 2024). Several interviewees expressed hope that *Dr Deaf* might serve as an adaptable model that could be replicated or reimagined in other regions by deaf academics in those regions, to reduce such inequities. Interviewees also expressed concern about the underrepresentation of certain academic fields. While the social sciences and humanities were well-represented, those from STEM disciplines hoped to see more deaf scholars from their fields included in future editions of *Dr Deaf*.

Even among those who *could* attend, access to resources was not always equal. For example, participants from several southern European countries reported little to no interpreter provision at their home universities, and when available, interpreters often lacked fluency in English. These disparities shaped participants’ feelings of belonging or disconnect in *Dr Deaf* spaces. Naiara, from Spain, remarked: “Sometimes I see something during a discussion, and I think ‘all very nice, but this doesn’t apply to the Spanish situation at all.’”

### *Dr Deaf* as a physical space

**Figure 2:**
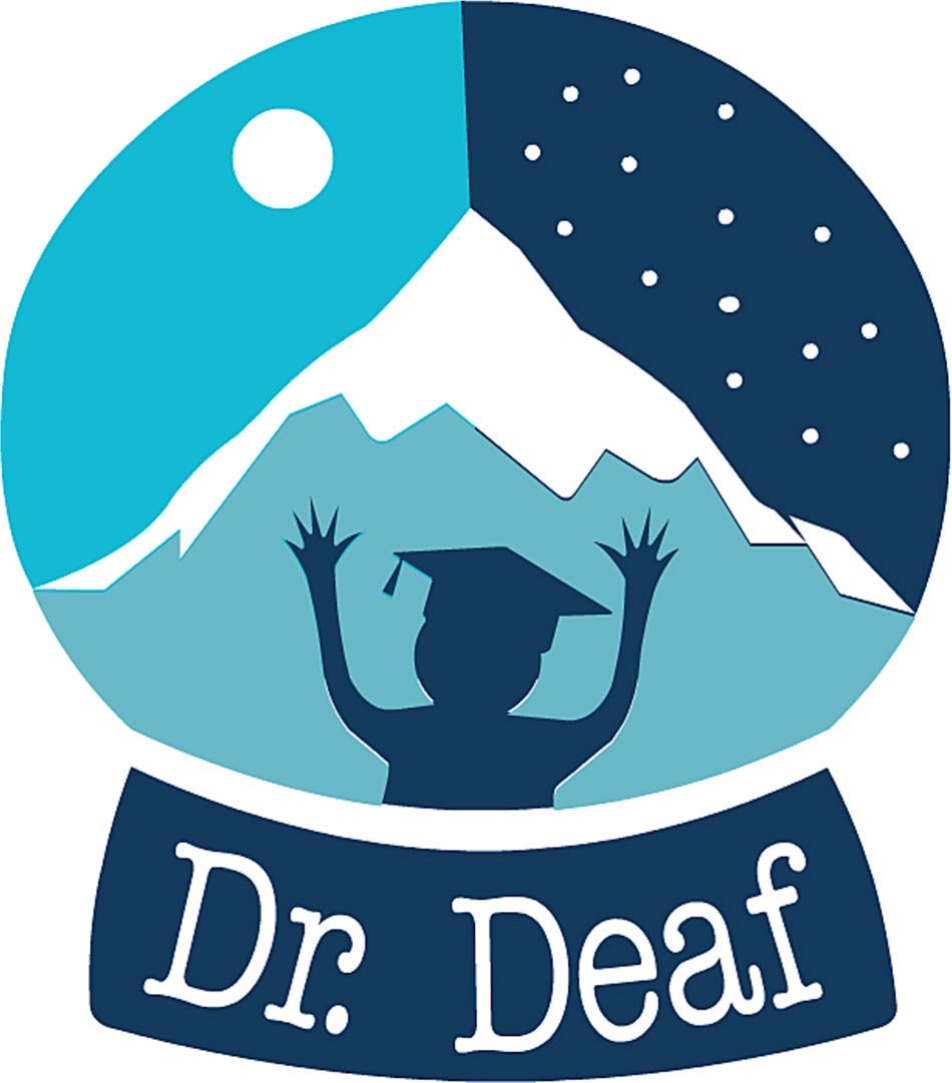
*Dr Deaf* logo. The official *Dr Deaf* logo, depicting a silhouette with raised hands in front of a mountain under a day–night sky, with the text *“Dr. Deaf”* beneath. Logo designed by Marta Morgado.

The physical setting of Ål Folkehøyskole, where *Dr Deaf* sessions are held, plays a vital role in shaping the overall experience. The schedule is designed so that most participants arrive together on the same train from Oslo. For many of the participants, the deaf academic space thus begins to take shape during this shared journey: the 3-hr train ride provides time to chat, reconnect, or get to know each other. Upon arrival, participants often comment on the beauty of the mountain landscape, describing a sense of calm and feeling “away from it all.” This setting shapes the experience of arrival—marking entry into a deaf-centered space where people could pause, connect, and engage fully. For many, the surrounding mountains, which feature in the *Dr Deaf* logo (see Figure 2), offer both a visual backdrop and a sense of distance from their everyday lives. For some, their biggest challenge therefore came *after* the workshop: transitioning back to hearing academic environments. Naiara: “Actually it’s a good thing the Ål school is so far away. On Friday I always feel time is closing in, and then there is the trip to the train station, the three-hour train journey, the plane, etc.—a lot of time to get home. That helps with the transition.”

Participants and teachers pointed to the compact and immersive nature of the campus as essential to the sense of community that has developed. The layout of the space where the sessions are held—spread over just two floors or sometimes even one—facilitates interaction and informal engagement. Alicia, a teacher, contrasted Ål with larger institutions like Gallaudet University, noting that at Ål, “everyone is on two floors. If I want to check out what someone is doing, I can just walk around the corner, and if they’re free, I can start a conversation.”

The linguistic and spatial availability also allowed for a level of spontaneity and serendipity rarely found in hearing academic environments. Ingela: “you can spontaneously meet people—or decide you don’t have that much in common after all. In hearing academic spaces that is much harder because working with interpreters doesn’t allow for a lot of serendipity.” Octavian described the closeness created by the setting more playfully: “There is no escape—the space means we are close together for five days, while in a larger place the network would be more diffuse.”

Robert, one of the teachers, agreed that what makes *Dr Deaf* special is not just the program but the shared physical space and being there around the same goal. The fact that participants and teachers live, eat, and attend sessions together creates opportunities for constant mingling, shared storytelling, and ongoing calibration between different signing styles and backgrounds. “We learn where people are from, their backgrounds—that’s so different from a conference. It helps with calibrating our teaching to the participants,” he added. Ingela also highlighted the importance of the after-workshop activities, such as the llavo (a gathering around a fire for a barbeque), or the traditional venue Gamle Stua (an old cabin at the site), as essential components of the social fabric of *Dr Deaf*. These moments between formal sessions are where much of the bonding and informal learning occur.

At the same time, the constant mingling can be taxing for participants and especially for teachers (who also must prepare sessions), and the physical space of Ål Folkehøyskole also presented challenges for some deafblind and deaf-disabled participants. The folk high school where *Dr Deaf* takes place is in a hilly, rural area of Norway. In winter, thick snow can make it difficult to move between buildings, especially at night when it is dark. Cabins are scattered across the campus and often require walking on uneven paths. The nearest shop is only accessible by car. In winter, at some outdoor evening activities, light conditions are not optimal.

### *Dr Deaf* as a safe space

Another frequently mentioned aspect of *Dr Deaf*, both by participants and teachers in the study, was that they experienced it as a safe space. Before going on, we want to reiterate that our positionality may have influenced interviewees to withhold accounts of times when they did *not* experience *Dr Deaf* as a safe space. We have heard anecdotally from some participants about such moments—for example, discomfort as a brown or black person in a majority-white space, or a deafblind person being tired of asking others to accommodate. For many participants in the study, though, *Dr Deaf* provided a safe space—emotionally, intellectually, and socially—a space in which they felt understood and not judged. Phoebe: “*Dr Deaf* gave me a space to process things, share my experiences, without anyone judging me. I felt like people understood me and knew what I was trying to say.” For Kali, a participant from computer science, *Dr Deaf* created the kind of intellectual safety that allowed her to engage beyond her disciplinary boundaries, describing it as a space where she felt comfortable asking questions outside of her field—something she says she would never do elsewhere. This safety extended to classroom dynamics. Teachers observed that during informal drop-in sessions, participants often asked fundamental questions that they had previously felt ashamed to ask, such as the difference between quantitative and qualitative research. Katie, a teacher, explained: “*Dr Deaf* gives them a space where it feels safe to ask, to admit you don’t know something. The deaf signing space allows for this, it’s smooth and comfortable.”

Importantly, participants stressed that this safety was not about lowering expectations. Rather, *Dr Deaf* was described as a demanding space—in a way that was often affirming and collectively accountable. Active effort was required from everyone, such as in navigating differences in signing fluency, language, and cultural backgrounds, and academic experiences. Participants acknowledged that building mutual understanding required work—and it was a kind of work they were willing and eager to do. We are aware from evaluation sessions and informal feedback that for some, this effort was much greater—for example, deafblind and deafdisabled participants, first-time participants beginning to build networks, and participants who are naturally more introverted.

We saw a tendency for participants to link the above-discussed sense of safety to not needing to filter or mask their experiences for *hearing people*, allowing for open conversations about structural inequalities in academia. Octavian highlighted how *Dr Deaf* allowed for discussions that would be impossible under the “hearing gaze”:

*Power differences, audism, ableism—it’s impossible to have the same kind of discussions when there are hearing people in the room. At Dr Deaf, you don’t have to temper down your words, your emotions, your facial expressions, your body language. It’s a celebration of deaf ways of being and deaf knowledge.*

Peter framed this in terms of “masking”:

*Dr Deaf is a space where deaf academics* can *take off their shields. The shields are hearing expectations of deaf people—how we’re supposed to behave, talk, even our humour. We* can *take those off and just be ourselves. You’re still an academic—we don’t just toss that away. The formal deaf academic discourse is there. But we* can *be deaf academics.*

Peter and Octavian were talking about the contrast between deaf and hearing academic environments. We acknowledge that participants still may filter and mask in relation to other factors such as race, gender, and neurodivergence.

*Dr Deaf* also created a space to explore emotional vulnerabilities often hidden in other academic settings. For instance, it made it possible to talk about impostor syndrome in academic environments (Chua et al., 2022). Nora, a junior teacher, was surprised to hear that even senior teachers still struggle with it. She said to feel confident that she had been asked based on her skills and contributions, not merely because she was deaf: “Tokenism is not a concern for me in *Dr Deaf* or other deaf academic spaces, whereas in hearing academics spaces I would wonder about this.” Not all teachers shared the same feelings about this though. Okan, a teacher from Türkiye residing in Germany, described impostor feelings during their first days at *Dr Deaf*:

*“Why me?” they asked. “There are good academics. Why me? I come from a different identity, experience, philosophy, perspective, and language background compared to many of them. Many are more privileged, Western, and know English well. That is certainly a point of comparison in the deaf academic community. I thought maybe the participants and teachers would think ‘Okan doesn’t know English well—I* can *learn more from someone who has published in English a lot’. Maybe I’m wrong! I was having that feeling of insecurity, but I’m not sure if it was about me and/or about them.”*

Although not explicitly addressed in the data, this sense of tokenism and impostor syndrome may still be the case for some teachers. These reflections suggest that while the values of *Dr Deaf* were to foster openness and validation, underlying hierarchies of privilege, language, and academic capital shape how this space is experienced and negotiated. This process can be deeply uncomfortable and, for some, bring a strong sense of difference, which they work through and/or struggle with alongside their teaching and participation.

### *Dr Deaf* as an interpreter-free space

*Dr Deaf* was often described as an interpreter-free space, even though deaf interpreters are sometimes present to work with deafblind participants. When people referred to it as interpreter-free, they actually meant the absence of hearing interpreters facilitating access between signed and spoken languages. Alicia shared that she tends to be quieter at hearing conferences, participating in one-on-one conversations, but rarely speaking up in a larger group. In contrast, at *Dr Deaf* and other deaf conferences, she felt more assertive and outgoing. When asked why, she pointed to the lack of interpreter alignment and the feelings of impostor syndrome that this led to. She recalled a painful interpreting experience at a major hearing academic conference where, after her presentation, her PI had to step in and ask the audience to disregard the bad interpretation, saying, “Alicia actually knows her stuff.” But by then, the damage had been done—no one asked her questions. At *Dr Deaf*, the dynamic was reversed. She felt seen and recognized, able to engage on equal terms, without the need to constantly prove her expertise through someone else’s voice.

Interview participants all described the experience of working with hearing interpreters in “hearing” educational settings as exhausting and alienating. While participants and teachers in the study are happy to calibrate their signing to accommodate other participants—and this is indeed seen as integral to international deaf spaces (Moriarty et al., 2024)—calibrating their signing for interpreters is something they were generally less happy to do. Kali: “No interpreters means I can throw off the interpreter calibration gear.” Adjusting to working with interpreters goes beyond feeling tired and alienated, as it can impact academic participation over time. Robin recounted:

*When I go to Dr Deaf I remember how I was at school. We were taught directly in sign, no interpreters, and some of our teachers were deaf. I was always the one raising my hand, asking questions, wanting to contribute. But this changed when I started university—I stopped doing it. Mainly because of the interpreter. Whenever I asked a question, the room would fall silent – even when the interpreter translated it correctly – or the interpreter wouldn’t understand me, and I had to spell everything out. After a while, I just gave up.*

Teachers similarly described the challenges of teaching hearing participants with interpreters. Peter: “I can’t monitor the participants’ understanding—I need to trust the interpreter. Teaching directly in sign, it’s much easier to see: am I clear? Do I need to repeat myself?”. This again connects to the notion of “deaf rhythm”—the presence of interpreters introduces, among other things, lag time, which can disrupt the natural flow of interaction and affect teachers’ and participants’ ability to monitor understanding, take turns fluidly, and offer requests for clarification in real-time.

For sighted deaf teachers, teaching without interpreters allowed for more spontaneous, responsive, and reciprocal interactions. Deaf rhythm extended to preparation time—preparing for a session when working with interpreters differs from preparing for a *Dr Deaf* session. Nora added: “With interpreters, you need to send prep material way in advance—which I hate, because you can’t change things last-minute. It removes flexibility. At *Dr Deaf*, it’s more responsive.” Okan added that this also changed the relational dynamics of the space: “without an interpreter, there is a greater sense of comfort and confidence in communication.”

Despite this, the interpreter theme is not entirely absent from *Dr Deaf*. One of the most popular workshops during the PhDeaf 2024 workshop was precisely on how to work with interpreters, with an emphasis on deaf academics exchanging their own experiences, strategies, and know-how.

The celebrated flexibility of “no interpreters” in the *Dr Deaf* space—while liberating for many sighted participants—does not always translate into the same kind of autonomy for some deafblind participants. They often bring their own interpreters/guides, whom they must prepare in advance and sometimes fund themselves (not all governments cover international expenses). These interpreters are usually present not only during teaching sessions but also during meals and social activities (even when not actively working), adding layers of logistical and emotional labor for deafblind participants. This presence introduces a complex dynamic: as deaf interpreters, they often take part in deaf spaces, may know some participants personally, or may want to network and engage. Yet these interpreters are not participants in the same way, and their role positions them differently within the space. This positioning requires ongoing reflection and awareness about how freely they can mingle without compromising the involvement or comfort of the deafblind participant within the group.

### *Dr Deaf* as an IS space

One of the distinct features of *Dr Deaf* is its use of IS as the primary language of instruction and interaction. Participants come to *Dr Deaf* with varying skills in IS, though many seem to have at least *some* experience. We have also noted that having one or more people from the same country who are more confident in IS often helps encourage participation.

Many participants in the study described IS as a shared, visual language fostering a sense of inclusion and accessibility across linguistic and national differences: “a language we can all join” (Robin) or “all understand” (Maija). IS was described as a way of communicating that is “easy to learn” or can be intuitively understood without a learning process (Kusters, forthcoming). Participants highlighted a rapid learning curve over the course of the workshops—not just in themselves but in their peers as well. The intensity and speed of getting used to communication in IS is another aspect of deaf rhythm specific to international deaf space. Maija, who came to the workshop with a lot of IS experience, said:

*Yes it* can *be challenging for new signers of IS, but at the same time I* can *see they are really motivated, and they feel they* can *learn. No one is saying “I can’t do it,” and I always see progress. Dr Deaf offers a safe space to learn and use IS.*

Kali, who came to *Dr Deaf* from the US and had experience with both ASL and Chinese Sign Language, described the steep learning curve she faced as a newcomer to IS:

“The first *Dr Deaf* workshop was my first ever IS experience! I was nervous. But the poster said that new IS signers were welcome, and that helped me feel like I could try. IS borrows a lot from ASL, so that was okay for me—but I tried to include Chinese signs too.” Kali described the learning curve as both intensive and fast, yet also developing over multiple periods of participation in *Dr Deaf*: “I could follow what others were saying but producing IS was harder. At that first workshop, I spent most of my energy just figuring out how to express myself and how to communicate. But by the second one I could focus more on understanding the content, taking notes, and participating.”

While the challenges of using IS—especially for new IS signers—were acknowledged, participants who already had experience with IS or at least *motivation* for the use of IS overwhelmingly preferred this to interpreter-mediated interactions.

In addition to knowing ASL, as mentioned by Kali, knowing English and being able to lipread in English was described as helpful to understand IS. But even when English literacy was needed to support comprehension (e.g., to follow PowerPoint slides), participants in the study reported that for them, working in IS was more empowering than trying to follow a hearing teacher through an interpreter in their national sign language.

Teachers tried to be mindful of the consequences of ASL or English use within IS or alongside IS and adapted their pedagogical strategies to the IS environment. Octavian explained:

*We’re committed to making this an international space. That means ditching the [English] fingerspelling as much as you* can*, and embracing visual explanations—show, don’t tell. It helped me to practice in advance, stay open for student feedback, and learn from their signing too.*

This included being mindful of how to respond to participants’ questions: “if someone asks me a question in ASL, I make sure not to respond in ASL back. It’s about the whole group, not just one person.” Thus, while calibration could occur at the individual level, such as in one-to-one conversations, it also could take the broader collective space into account.

The shift from fingerspelling and lexical borrowing to more visual, conceptual signing was often seen as a sign of communicative maturity in IS but also as a sign of deaf academic maturity. Reminiscent of Albert Einstein’s phrase “If you can’t explain it simply, you don’t understand it well enough,” Robin elaborated:

*If you’re a hearing academic and you’re writing complex sentences you’re often seen as a good writer. In IS, it’s the opposite. If you can explain something simple, clearly and visually, that makes you a good IS signer, and it means you know what you are talking about.*

*Dr Deaf* teachers were often recognized for their ability to work in IS at a high level, particularly in using expansive visual explanations. Maija noted: “Some people, when they use IS, they really change and explain things in a more visual and expansive way. It’s a skill not many people have.” Octavian described IS not only as a teaching tool but as a site of creativity and intellectual play: “I appreciate the intellectual creativity of IS. It’s fun. It’s a challenge I’m glad to accept.”

IS was also seen as an inclusive practice, with teachers and participants sharing the same communicative space. Naiara described IS as a moral orientation (see Green, 2014; Moriarty & Kusters, 2021) rooted in deaf lived experiences grounded in a mutual commitment to meaning-making: “Calibration has always been a part of our lives. If we don’t understand, that’s fine— we’ll do the work.”

Ingela, a teacher and former participant from Sweden, actually felt limited, said that if she could use Swedish Sign Language, she would feel much more comfortable, could build in more nuance. But at the same time, she said she felt that students were very accepting and respectful of the limitations she felt.

*One comparison: we had a summer school here in Sweden which we co-organized with RIT. There were 10 to 12 [mostly hearing] students joining from the US. I feel those students are much less aware of the need to calibrate. They just sign ASL and think everyone will understand. The Dr Deaf experience was very different—I felt there was a mutual willingness to understand each other, asking for clarification—there was a much more positive and supportive vibe.*

However, this is not to say IS was always experienced unproblematically by all participants. Hilde noted: “when IS *is* an issue, we don’t always say so,” pointing to the risk that the moral orientation toward using and appreciating IS can start to feel like an imperative.

For those deaf academics who have attended local hearing schools without access to sign language and may only begin learning their national sign language in (young) adulthood, engaging in international deaf spaces—where IS is often the default—can pose an overwhelming learning curve. These spaces can feel inaccessible (Kusters, forthcoming) and, as a result, people with these profiles may choose not to attend at all.

In addition, IS has evolved over the years, and its use has become faster and more condensed, especially among experienced signers who have long participated in transnational spaces. This speed can be overwhelming for those encountering it for the first time, who not only have to grasp unfamiliar lexicon but also decode rapid signing in real time. During meals and breaks, there is time and space to calibrate—to slow down, to add extra explanations, and to learn each other’s signs for specific concepts, without the time pressure that comes with workshops and presentations. In contrast, during workshops and presentations where one person is signing to a group, understanding tends to vary more. As a result, a number of participants have found it difficult to follow presentations and workshops. Questions for clarification are welcome, and participants often broker for one another—for example, by re-signing an IS utterance in a different way, or by translating it into another language, such as when a Turkish participant quickly translates something into Turkish Sign Language for another Turkish participant. However, deaf participants who are new to IS can feel hesitant to ask for repetition often, fearing they will stand out or slow down the group (Kusters, forthcoming). This creates an additional layer of pressure that can impact participation, feelings of belonging, and confidence.

### *Dr Deaf* pedagogies/teacher strategies

Teachers brought a wide range of strategies to their sessions, many of which were shaped by the specific demands and affordances of a deaf multilingual environment. A consistent theme in the discourse about this was the importance of interaction and responsiveness. Kate and Robert described it as a core strategy to begin by checking what participants already knew and inviting them to contribute examples from their own experiences. Other strategies emphasized barriers as well as benefits in relation to using written English. Nora noted that because English text on slides could present barriers, it was better to minimize written content and to link keywords in English to key signs. However, she noted that more expansive text on slides can also help: “it helps people avoid writing loads of notes. So we need to balance it.” Most teachers gave participants time to read what was on a slide before signing—aligning with the principles of deaf rhythm.

Robert described a deliberate move away from lecture-style teaching, having experienced teaching at both UCL in the UK and Gallaudet in the US:

*At UCL it was more “lecture lecture lecture” while at Gallaudet it’s “talk talk collaborate.” So I had to learn different teacher strategies. At the PhDeaf workshop I’d explain the topic, say why it matters, give an example, and then hand it over to the participants.*

He also emphasized the value of limiting each session to one or two key points, allowing for discussion and reflection—these back-and-forth interactions being a core aspect of deaf rhythm. This is reminiscent of what Mette shared about participants being sources of deaf know-how/epistemologies themselves, knowledge that could be transferred to other participants, such as by way of workshop contributions, mini-presentations, and panels.

For most teachers, the *Dr Deaf* pedagogy extended beyond technique. Ingela reflected one of the most important things was being open: “share experiences, including the not-so-nice aspects of academic life—like manuscript rejections.” Hilde agreed: “I show the good and the bad days. Show that I have been a BA and MA student before. (…) it’s important not to show off, but to show how to navigate deaf academic life—the successes and the failures.” This storytelling approach created a space of mutual exchange. As Robert explained, “At *Dr Deaf*, sharing personal experiences validates them—people want to hear each other’s stories. It creates a shared understanding.”

This practice created pressures as well. Robert and Okan both acknowledged the vulnerability involved in teaching deaf participants as opposed to (most) hearing participants, admitting to being more nervous teaching deaf participants precisely because of the deeper investment and expectations. They both phrased this almost exactly in the same way: “You know you’re being a role model. You must open yourself in ways you don’t do for hearing participants—that’s not always easy to do.”

At *Dr Deaf*, teaching was also seen as revealing what is often hidden for deaf scholars in academic life because of language barriers. “We make the invisible visible,” Peter explained. “We expose the ‘hidden’ secrets of academia. We give participants the tools to be more successful.” This relates to a comment made by Hilde about *Dr Deaf* offering incidental learning that is often missing in mainstream environments. “It provides a glimpse into academic life, so [prospective PhD] participants can see if it fits them or not.” PhDeaf is an important space for MA participants to consider if a PhD is something for them or not. Also, by attending PhDeaf they sense that they will not be alone and that others have built up this shared pool of deaf academic knowledge, to which they will have access. Hilde and Peter both noted that hearing students often gain this kind of informal exposure through networks, discussion groups, and academic activities in their universities, while deaf students frequently miss out on this because of the access labor involved.

### What teachers gain from *Dr Deaf*

While much of the focus of *Dr Deaf* is on supporting and empowering participants, teacher interviews revealed that the experience can be equally meaningful for them. Teaching at *Dr Deaf* was described as a professional responsibility and, by most teachers, also as a space of personal and professional growth. All teachers expressed feeling honored to be invited to join the *Dr Deaf* teaching team, and despite their busy academic schedules, many accepted the invitation without hesitation. Hilde clarified that she doesn’t simply say yes to every opportunity: “When it is about supporting other deaf scholars, I say yes almost immediately. My heart is more into helping deaf participants—for hearing participants, I’d be more selective, think about whether I have the time.” Teachers mentioned it was rare to work with other deaf teachers or to teach deaf students in a fully signing, interpreter-free space. Outside of *Dr Deaf*, most *Dr Deaf* teachers primarily or only teach *hearing* students, often through interpreters, often in programs for sign language learning and sign language interpreting training (a common experience for deaf academics, at least in Europe).

Teachers expressed a strong sense of purpose and responsibility to “pay it forward,” either because they had once received such support themselves or because they had lacked it and wanted others to benefit from it. Nora, who very recently (2024) obtained her PhD commented: “I got a lot of support as a student, and now it’s my turn to give,” while Hilde obtained hers in 2012:

*I am among the first generation of deaf academics [with PhDs, in Europe]. That time, there weren’t that many other deaf academics I could talk to. [...] I often felt lonely, and it had a serious impact on my mental health. That’s a reason why I want to give back to the current generation of deaf students. To tell them that frustrations are a normal part of life and of doing a PhD—but also to tell them: “you are not alone.” If I knew that back then, my academic journey would have been very different.*

Hilde reflected that one of the benefits of teaching at *Dr Deaf* is also to collaborate across generations and career stages. Ingela agreed: “It broadens my world. I get to see the current generation of deaf PhD students, and what they are working on.” Several of the current deaf PhD students who participated in the study expressed the same desire to “give back” and share what they gained. Naiara: “Many deaf participants from the first *Dr Deaf* generation will soon finish their PhD—we also need to think about how to give back to the community.”

While all reported feeling honored to be invited and committed to the program, teachers also acknowledged the intensity of the workload as they took time out from their universities to teach at *Dr Deaf*. Nora and Mette recalled struggling during the first PhDeaf workshop they taught, as they had been teaching in their own universities up to that point. “I had no time to prepare, no space to get ready in advance,” said Mette. Social events, often a valued part of the *Dr Deaf* experience, could be overwhelming for teachers as well, noting that being “socially available” all day could be exhausting. “Sometimes it’s too much for me,” Mette admitted. “It can be draining. We need strategies to give us breathing space—like sending participants out [for group work] with a topic to work on.”

While teachers acknowledged the additional labor involved in preparing for and teaching at *Dr Deaf*, they distinguished this from other kinds of deaf labor, which can be exhausting or tokenistic, and are therefore often referred to as “deaf tax” (Aldalur et al., 2022). Okan saw it as “two-way traffic,” a rare instance of additional deaf labor that was “worth the effort, worth my time.”

For Octavian, *Dr Deaf* provided not only a space to share knowledge but also one of moral and intellectual support: “I gain tools, conversations, and insights that help me not just as a scholar, but also as a teacher, advisor, and mentor.” He, along with others, emphasized the value of navigating complex academic processes—such as publishing—within a collective of deaf peers. What he called “collective wisdom” of *Dr Deaf* was seen as a vital resource that newer participants, including junior faculty, could draw on.

### *Dr Deaf* and inclusion: the role of general education systems

When interviewing teachers at *Dr Deaf*, one of the issues that came up was whether *Dr Deaf* might inadvertently be taking over responsibilities that should lie with universities—specifically, in providing appropriate support, training, and mentorship for deaf students. While this is about academic training, it is also about the role of general education systems in providing inclusive learning and training experiences for deaf academics, both at the MA and PhD levels and for deaf early career scholars. Responses varied, reflecting a mix of concern, pragmatism, and recognition of structural limitations.

Some teachers expressed frustration with universities when seeing how unprepared many participants were when they arrived at *Dr Deaf*. Some pointed to lack of inclusive education at the postsecondary level: Katie:

*Many of them didn’t know things they really should know already [about research design]*—*things their universities and supervisors should have taught them. Some of these participants are paying out of pocket to attend. Universities should be more aware of this. Maybe it’s a strong way to say it, but we’re kind of repairing the education system.*

Hilde confirmed this: “I was surprised about the gaps in knowledge and the superficial understanding about academic life of some participants: what supervision means, that the writing process can be hard, knowledge about theoretical frameworks and how to apply them.”

For them, *Dr Deaf* was stepping in to fill gaps left by the general education system (not just higher education) that is failing deaf participants. Crucially, the experience at *Dr Deaf* helped some participants identify and articulate their access needs, although they knew those specific needs could maybe not be filled in by universities alone. Franklin recounted: “My university asked me how they could support me, but I didn’t know what to ask for. I knew something was missing but I didn’t know what or couldn’t explain. When I went to *Dr Deaf* I realized: this is what I was missing.” John said he had a supportive mentoring program at his home university for new tenure-track faculty, but *Dr Deaf* still provided him with something his hearing support network could not. He contrasted his experience with another deaf faculty member in his country who ended up dropping off the tenure-track because they failed to understand the expectations for tenure-track faculty that he received at *Dr Deaf*. John: “*Dr Deaf* taught me what is expected from academics.”

John’s experience shows that even when universities offer support, there is still something that *Dr Deaf* offers in addition. Nora: “Even if that problem were solved, we would still need *Dr Deaf* as a breathing space. It’s not meant to resolve everything. We would still need deaf spaces.” Peter offered a broader perspective, noting that many minority academics—first-generation scholars, women, people of color, disabled academics—benefit from targeted support structures that universities have developed. “Universities do design this for hearing minority faculty,” he said, “but it’s not designed for deaf academics. *Dr Deaf* provides the deaf part of this training. The universities often don’t have the resources for that, so we provide it instead—and that’s fine.” At the same time, he expressed positive surprise at the number of participants and the quality of their contributions. For him, this was evidence that there are now more opportunities for deaf students to succeed in higher education than there were 20 years ago. This observation suggests progress, even if uneven, in deaf people getting bachelor’s degrees and being able to be accepted to graduate programs.

Robert offered a reality check: “*Dr Deaf* is great, but let’s be honest—it doesn’t solve everything in academia. There are still no proper pathways or job opportunities for deaf scholars.” This comment highlights the broader structural challenges that deaf academics continue to face, beyond access and training.

Teachers thus considered the dual nature of *Dr Deaf*: as a space where deaf participants and researchers could get critical training that they often failed to get in their home university settings, but also as a breathing space, both in terms of regaining energy for their home universities and for expanding their identities as deaf researchers.

## Discussion and Conclusion

*Dr Deaf* enacts deaf epistemologies and pedagogies in practice (Kusters, 2017; Ladd, 2022). Participants do not simply *learn*—they co-construct deaf academic ways of knowing, and, in doing so, engage in *academic becoming*: coming to see themselves—and be seen by others—as academic actors. Some access imagined deaf academic futures; others (re)align identities as both researchers and deaf people. The frustrations of learning to “play the game”—understand the codes, absorb tacit knowledge, and become part of a community—are not a uniquely deaf experience, and resonate with those of first-generation and other minoritized academics (Addison, 2016; Bravata et al., 2020; Mullen, 2016; Robinson & Henner, 2018). Importantly, many deaf academics are also multiple minoritized, and their experiences are shaped by intersecting forms of marginalization (Garberoglio et al., 2019; Hou & Moges, 2023; Stapleton, 2015; Hou & Ali, 2024). What we emphasize here as a distinctive, though not exclusive, feature of *deaf* academic becoming is the experience of linguistic and communicative exclusion: many signing deaf academics work in environments where communication is almost always mediated. Academic becoming also requires unlearning deficit framings—for example, assuming reviewers are hearing by default. In addition, what is distinctive about this space of deaf academic becoming is its international nature, where people extend their small national pools into larger international and intercultural networks.

Linguistically, IS is approached not only as a suitable lingua franca but also as a moral practice of effort, mutual accommodation, and calibration (Moriarty et al., 2024; Moriarty & Kusters, 2021), countering national educational systems that often expect deaf students to adhere strictly to standardized spoken languages, or to national sign languages to interface with interpreters (Russell, 2021). Pedagogically, *Dr Deaf* combines vertical instruction with horizontal mentorship. Teachers return as participants, and participants become teachers, “paying it forward” to new generations of participants, and responsibility moves across stages. As one teacher, Katie, noted, it also reconfigures whose knowledge and expertise is being valued:

*in education, we often hear that deaf people have low reading levels, and no future. But here, they see deaf academics who are just as smart as hearing ones. In the future, we need to stop asking hearing teachers of the deaf for advice. Deaf academics are the real experts.*

We conceptualize these shifts as *realignment*—structural, pedagogical, affective, and linguistic. Structurally, the usual dynamics of higher education are inverted: deaf academics teach deaf students, directly in IS. In this way, *Dr Deaf* aligns methods of teaching and learning with deaf ways of knowing. The materiality of Ål—the deaf-owned infrastructure and surrounding mountains—although it came with limitations for some participants, enabled the rhythm, calibration, and informal learning that would have been harder elsewhere. The notion of a (admittedly deaf-sighted-centerd) *deaf rhythm* captures this space and flow: language-concordant teaching, interpreter-free exchanges, the back-and-forth interactions in the classroom, and the informal conversations over meals.

At the same time, this alignment was not universal. Newer IS signers, participants from non-English speaking writing traditions, and those with additional disabilities, reported moments of disconnection or fatigue. Access to higher education, interpreters, travel funding, English and IS proficiency, and national networks that connect participants to *Dr Deaf* shaped who could attend and to what extent—privileging already privileged deaf people and reflecting broader inequalities in higher education. Even so, participants came, and some returned. This is not necessarily because *Dr Deaf* is an ideal fit for everyone but because they may not be able to afford the luxury of choosing between deaf academic spaces, and so gravitate towards what is the best option, if not the only one available to them. This makes the need for more—and more diverse—deaf academic spaces all the more urgent.

Importantly, *Dr Deaf* is not perceived merely as “filling gaps” left by universities. While it provides belonging and language-concordant education—elements too often absent from participants’ higher education experiences—participants also viewed *Dr Deaf* as something *qualitatively different.* Not a supplement to mainstream institutions, but an alternative that operates on different terms. Against the “illusion of inclusion” of interpreter-mediated access, *Dr Deaf* offered a model of access grounded in deaf values: collectivity, calibration, mutual responsibility, and shared vulnerability. It is not a wholesale solution but part of an ecology of support. This also points to the limits of one-off interventions. Okan questioned why such alignment should wait until late-stage PhD trajectories—why not embed deaf-led, deaf-centered spaces earlier across educational trajectories?

A practical tension concerns scope. We have received suggestions to open *Dr Deaf* to non-academic members of deaf communities and to “bridge gaps” between deaf community spaces and academic spaces. Deaf communities have been conceptualized as collective in nature, shaped by shared experiences of oppression and linguistic and cultural affirmation (Ladd, 2003). Within this collectivist orientation, there is often a strong emphasis on building bridges between deaf academia and broader deaf communities (O’Brien, 2017) and on the responsibility of deaf scholars and other privileged deaf people to “give back.” This mirrors broader expectations placed on minoritized scholars more generally: performing academic excellence while at the same time being “of service” to their communities. We share this commitment, and each of us is actively engaged in research dissemination and public scholarship that reaches wider audiences. At the same time, we caution against what we term the *flattening of deaf spaces*: the egalitarian pressure to make every deaf space serve everyone in the community, regardless of its original aim. Flattening occurs when a space designed to meet the needs of a specific deaf group—in this case, deaf academics—is stretched beyond its original purpose, often due to internal and/or external expectations of being accessible to all community members. This can dilute the very conditions that made the space effective and meaningful in the first place. We argue that there is value in allowing specific deaf spaces to remain specific: environments that make room for particular forms of deaf epistemological, pedagogical, and professional development. While they should remain connected to deaf communities, they should not be expected to serve everyone in a community.

The practices described here—centering deaf epistemologies, cross-stage mentoring, fostering linguistic calibration through IS, and cultivating a deaf rhythm—offer a flexible framework for similar initiatives elsewhere, including in Majority World contexts where deaf academic infrastructures are sparse. These values also have implications for deaf education more broadly. They should not be limited to those who can enter academia in the first place and should be part of the educational experiences of deaf children and young people as well. While the exact form and language use of such spaces will vary across contexts, the values underpinning *Dr Deaf* offer a flexible framework for creating educational environments built on deaf terms.

We close with limits and possibilities. *Dr Deaf* cannot include those excluded from academia to begin with, nor can it erase structural barriers participants face beyond (and sometimes within) the space. Its dynamics are shaped by white, English-centered, European/North American traditions, and the very rhythm that enabled connection for some may not for others, such as some deafblind participants. Yet, briefly and imperfectly, *Dr Deaf* interrupts deaf-hearing inequalities and makes tangible alternative ways of knowing and being a deaf academic. As the number of deaf scholars grows across disciplines and geographies, sustaining and diversifying deaf academic spaces will be all the more essential.

## Footnotes

1 This column reflects participants’ self-descriptions at the time of the interview. We recognize that race and ethnicity are complex, fluid, and context-dependent constructs, and that categories such as “Black,” “Asian,” or “White” may carry different meanings in different parts of the world. In some cases, broader categories (e.g., “White”) were applied based on dominant frameworks in academia; we acknowledge the limitations of such categorization.
